# Regulation of xanthine dehydrogensase gene expression and uric acid production in human airway epithelial cells

**DOI:** 10.1371/journal.pone.0184260

**Published:** 2017-09-01

**Authors:** Ryan D. Huff, Alan C-Y. Hsu, Kristy S. Nichol, Bernadette Jones, Darryl A. Knight, Peter A. B. Wark, Philip M. Hansbro, Jeremy A. Hirota

**Affiliations:** 1 Division of Respiratory Medicine, Department of Medicine, University of British Columbia, Vancouver, British Columbia, Canada; 2 Priority Research Centre for Healthy Lungs, Hunter Medical Research Institute, The University of Newcastle, Newcastle, New South Wales, Australia; 3 Firestone Institute for Respiratory Health – Division of Respirology, Department of Medicine, McMaster University, Hamilton, Ontario, Canada; National Yang-Ming University, TAIWAN

## Abstract

**Introduction:**

The airway epithelium is a physical and immunological barrier that protects the pulmonary system from inhaled environmental insults. Uric acid has been detected in the respiratory tract and can function as an antioxidant or damage associated molecular pattern. We have demonstrated that human airway epithelial cells are a source of uric acid. Our hypothesis is that uric acid production by airway epithelial cells is induced by environmental stimuli associated with chronic respiratory diseases. We therefore examined how airway epithelial cells regulate uric acid production.

**Materials and methods:**

Allergen and cigarette smoke mouse models were performed using house dust mite (HDM) and cigarette smoke exposure, respectively, with outcome measurements of lung uric acid levels. Primary human airway epithelial cells isolated from clinically diagnosed patients with asthma and chronic obstructive pulmonary disease (COPD) were grown in submerged cultures and compared to age-matched healthy controls for uric acid release. HBEC-6KT cells, a human airway epithelial cell line, were grown under submerged monolayer conditions for mechanistic and gene expression studies.

**Results:**

HDM, but not cigarette smoke exposure, stimulated uric acid production *in vivo* and *in vitro*. Primary human airway epithelial cells from asthma, but not COPD patients, displayed elevated levels of extracellular uric acid in culture. In HBEC-6KT, production of uric acid was sensitive to the xanthine dehydrogenase (XDH) inhibitor, allopurinol, and the ATP Binding Cassette C4 (ABCC4) inhibitor, MK-571. Lastly, the pro-inflammatory cytokine combination of TNF-α and IFN-γ elevated extracellular uric acid levels and *XDH* gene expression in HBEC-6KT cells.

**Conclusions:**

Our results suggest that the active production of uric acid from human airway epithelial cells may be intrinsically altered in asthma and be further induced by pro-inflammatory cytokines.

## Introduction

The airway epithelium is a barrier that protects lung from the external world we live in. Each day this barrier is exposed to over 10000 litres of air, which variably contains a complex mix of potentially pathogenic insults including allergens, air pollution, cigarette smoke, viruses, bacteria, and fungi[1–6]. In addition to acting as a physical barrier with tight junctions and mucus production[7], the airway epithelium is an active player in pulmonary immunity by sensing the external environment and responding with tailored activity. The airway epithelium senses the external environment in part through innate immune pattern recognition receptors that recognize pathogen associated molecular patterns (PAMPs) and damage associated molecular patterns (DAMPs) [1–6]. Following this sensing, immune mediators are secreted by the airway epithelium into the extracellular space to induce changes in the local environment, recruit and activate immune cells, and contribute to repair.

The complementary mechanical barrier and immunological properties of the airway epithelium are important for lung health, with dysregulation in these properties observed in chronic respiratory diseases including asthma [8–10], chronic obstructive pulmonary disease (COPD)[11–14], and cystic fibrosis[15–17]. The dysfunction of airway epithelial cells in chronic respiratory disease may be evident under basal resting conditions or under stimulation from agonists in the external environment, and genetic and epigenetic factors may also contribute[18]. Epithelial-derived cytokines and DAMPS that initiate early immune responses to environmental challenges are frequently dysregulated in asthmatics [19–24]. We have recently demonstrated that human airway epithelial cells are able to secrete the DAMP, uric acid [25], under basal and stimulated conditions[26]. The importance of this observation needs further exploration as uric acid has been linked to innate immune responses important in asthma and may be important in other chronic respiratory diseases[27, 28].

Production of uric acid results from metabolism of purines by xanthine dehydrogenase (XDH) [29, 30]. Evolutionary pressures in humans have rendered uricase dysfunctional, which is the enzyme responsible for further metabolism of uric acid into a more hydrophilic allantoin [31, 32]. The end result is accumulation of uric acid within the plasma (240–350μM) that is regulated by kidney tubular epithelial cells and urinary excretion[33]. From an evolutionary perspective elevations in uric acid must carry some net benefit to the host with the DAMP properties of this molecule offset by equally important biological function(s). A clear counterbalance to the role of uric acid as a DAMP [25, 34, 35] is the antioxidant properties of this molecule [36–38]. As an antioxidant, uric acid is capable of scavenging oxygen radicals and protecting ascorbate from oxidation by forming complexes with iron [36, 37]. The delicate balance of DAMP and antioxidant functions may be impacted by altered uric acid levels, which may contribute to chronic diseases [28, 39, 40]. In the respiratory tract, uric acid has been identified in both the upper and lower airways [41, 42], and is elevated following allergen and particulate matter exposure [28, 43]. The precise biological mechanism(s) that reconciles the dichotomy of uric acid DAMP and antioxidant functions remains to be determined, although a transition from soluble concentrations (anti-oxidant) to insoluble crystals (DAMP) may be important[35]. For these reasons it is imperative to understand the molecular mechanisms that regulate uric acid production and the cellular source(s) of this molecule with complex functions.

We have previously demonstrated that human airway epithelial cells express XDH protein and secrete uric acid under basal conditions *in vitro*[26]. We showed that ATP Binding Cassette Transporter C4 (ABCC4) is expressed in airway epithelial cells and contributes to extracellular uric acid transport[26]. Increased uric acid levels have been demonstrated in mouse and human lungs following house dust mite (HDM) exposure *in vivo* [28] and *in vitro*[26]. The mechanism by which HDM elevates uric acid production are unknown and may involve increased expression and activity of XDH, altered extracellular transport, cellular damage leading to release of intracellular contents, or a combination. Of these possibilities, regulation of XDH activity has been examined most extensively, revealing roles for diverse stimuli including iron, tumor necrosis factor-alpha (TNF-α), interferon-gamma (IFN-γ), dexamethasone, and hydrogen peroxide (H_2_O_2_)[44–48], all of which are relevant stimuli in chronic respiratory diseases.

Our overarching hypothesis is that uric acid production by airway epithelial cells can be induced by environmental exposures associated with chronic respiratory diseases. Using *in vivo*, *in vitro*, and clinical samples, we investigated the regulation of extracellular uric acid production by human airway epithelial cells. We first demonstrate that exposure to HDM, but not cigarette smoke, stimulates uric acid production *in vivo* and *in vitro*. We next show that human airway epithelial cells from asthma, but not COPD patients, have elevated levels of extracellular uric acid in culture. Extracellular levels of uric acid in airway epithelial cell cultures were sensitive to the XDH inhibitor, allopurinol, and the ABCC4 inhibitor, MK-571. Lastly, we show that the cytokine combination of TNF-α and IFN-γ elevate extracellular uric acid levels and *XDH* gene expression in human airway epithelial cells. Our results suggest that active production of uric acid from human airway epithelial cells may be intrinsically altered in asthma and be further induced by pro-inflammatory cytokines.

## Materials and methods

### Reagents

Freshly generated cigarette smoke extract conditioned media was prepared as previously described [49, 50]. Cigarette smoke from a University of Kentucky 3R4F cigarette was bubbled through 4 ml of serum-free medium, followed by filtration through a 0.22 μm filter, and adjustment with fresh media to an absorbance of 0.15 at 320 nm, to generate a 100% cigarette smoke extract solution. A 4% solution of cigarette smoke extract was made from the 100% solution for use in our experiments. Allopurinol, MK-571, dexamethasone, and cycloheximide were purchased from Cayman Chemical (Ann Arbor, Michigan, USA). Amplex Red uric acid analysis kits and epidermal growth factor (EGF) were purchased from ThermoFisher (Burlington, Ontario, Canada). Ferric ammonium sulfate and H_2_O_2_ were purchased from SigmaAldrich (Oakville, Ontario, Canada). Recombinant TNF-α and IFN-γ were purchased from Peprotech (Dollard des Ormeaux, Quebec, Canada). Bronchial epithelial cell growth media was purchased from Lonza (Mississauga, Ontario, Canada). Keratinocyte serum free growth media was purchased from Invitrogen (Burlington, Ontario, Canada).

### Human and animal ethics approval

All experiments were approved by the University of British Columbia’s Office of Research Services Ethics Committee and The University of Newcastle’s Animal and Human Ethics Committees. For human sample collection ethics approval and informed consent were obtained.

### *In vivo* mouse models

HDM exposure was performed with *Dermatophagoides pteronyssimus* extract (Greer Laboratories, Lenoir, NC) on female BALB/c mice, aged 6–8 weeks, purchased from Jackson Laboratory (Bar Harbor, ME). Mice were exposed to intranasal saline (35 μL) or HDM (25 μg in 35 μL of saline) under isoflurane anesthesia as previously described [51, 52]. HDM exposure was performed daily for 5 days, followed by 2 days rest, and repeated for 4 more days. Outcome measurements were performed 24h post-final exposure. Animal sacrifice was performed by terminal bleed via the descending aorta on isoflurane anesthetized animals. The ethics approval number for this study was A15-0189. Cigarette smoke exposure was performed on female C57BL/6 mice aged 6–8 weeks, purchased from the Central Animal House, The University of Newcastle, Australia. Mice were exposed to University of Kentucky 3R4F cigarettes or normal room-air using a custom-designed and purpose-build nose-only smoke system. Mice received cigarettes twice per day with at least 90 minutes of rest in between, 5 days per week, for 8 weeks, as previously described[13, 14, 53–56]. Animal sacrifice was performed by terminal bleed via the descending aorta on isoflurane anesthetized animals. The ethics approval number for this study was A15-0193. We used the Th1 dominant C57Bl/6 mice in our cigarette smoke model because this strain demonstrates an enhanced immunopathology and emphysematous phenotype[57]. We used the Th2 dominant BALB/c mice in our HDM model because this strain demonstrates an enhanced goblet cell metaplasia and airway dysfunction relative to C57Bl/6 mice in allergen exposure models[58]. For all animal studies, bronchoalveolar lavage (BAL) was performed on intubated animals with 500μL of sterile PBS, collected on ice, centrifuged to isolate supernatant, and supernatant archived for downstream uric acid analysis.

### *In vitro* airway epithelial cell culture models

Primary human airway epithelial cells were isolated from bronchial brushings from healthy subjects and those with physician diagnosed asthma and COPD based on ATS/ERS guidelines as previously described [59, 60]. Briefly, bronchial brushes were collected into 5ml of warmed bronchial epithelial cell growth media in a 15 ml conical tube. The brushes and media were vortexed for 30 seconds to remove cells from the brush. The brush was then removed and washed via pipette with the media in the conical tube to remove as many cells as possible. Cells were then centrifuged (1200 rpm for 7 minutes) for isolation, re-suspended in fresh bronchial epithelial cell growth media, and plated into 24 well culture dishes with 500μL of media. Subject characteristics are outlined in Table 1. Primary cells were grown under submerged monolayer conditions in bronchial epithelial cell growth media (Lonza—Switzerland).

**Table 1:** Clinical subject characteristics.

**Subject Code**	**Sex**	**Age**	**GOLD Stage**	**Predicted FEV1**	**Cigarette (Pack/Year)**
**Healthy Control Subjects**					
HC079	F	54	NA	105	0
HC085	M	47	NA	112	0
HC092	M	61	NA	96	0
HC080	M	61	NA	94	0
HC120	M	43	NA	117	0
**COPD Subjects**					
CA158	M	70	3	49	60
CA128	M	65	3	49	80
CA139	M	72	3	43	40
CA122	F	70	3	34	39
CA176	M	78	3	38	66
**Subject Code**	**Sex**	**Age**	**GINA Classification**	**Predicted FEV1**	**Cigarette (Pack/Year)**
**Healthy Control Subjects**					
HC066	M	65	NA	92	0
HC074	F	60	NA	116	0
HC082	M	52	NA	81	0
HC088	F	65	NA	NA	NA
HC089	F	69	NA	86	0
**Asthmatic Subjects**					
AS059	F	55	Severe	79	0
AS053	F	45	Severe	90	0
AS065	F	61	Severe	48	0
AS048	F	49	Severe	61	0
AS070	M	59	Severe	92	0
AS068	F	57	Severe	56	0
AS073	M	40	Severe	25	0
AS076	M	43	Severe	77	0
AS079	F	59	Severe	91	0
AS106	F	72	Moderate	81	0
AS087	F	67	Severe	36	0
AS088	M	47	Severe	46	15

To complement primary human cell experiments, we used a previously validated minimally immortalized human airway epithelial cell line (HBEC-6KT) grown under submerged monolayer conditions in keratinocyte serum free media with 50ng/ml of bovine pituitary extract and 0.6ng/ml of epidermal growth factor [26, 61–64]. All experiments with primary human airway epithelial cells and HBEC-6KT were performed at 90–95% confluency.

### Gene expression analysis

Total RNA was isolated using Qiagen RNEasy mini kits (Toronto, Ontario, Canada). For quantitative RT-PCR analysis, cDNA was generated using a high capacity cDNA kit (ThermoFisher—Life Technologies, Burlington, Canada). Quantitative RT-PCR was performed on an ABI StepOnePlus RT-PCR machine using PowerUP SYBR Green PCR master mix (ThermoFisher—Life Technologies, Burlington, Canada). *XDH* gene expression was normalized to *GAPDH* or *PPIA* based on our validation of a series of house-keeping genes in HBEC-6KT (data not shown) after reviewing suggested housekeeping genes for human lung epithelial cells[65]. qPCR data is represented as log2 fold change relative to control with statistical analysis performed on ΔΔCT values from independent biological replicates according to methods defined by Schmittgen and Livak[66].

The following primer sequences were used:

*XDH* Forward: 5’-CATGGGGAAGACAACCACAGG-3’,
*XDH* Reverse 5’-ATGGTCCTGATCCTGGCATCC-3’,
*GAPDH* Forward: 5’- ACGGGAAGCTTGTCATCAAT-3’,
*GAPDH* Reverse: 5’-CATCGCCCCACTTGATTTT-3’,
*PPIA* Forward: 5’- ATGCTGGACCCAACACAAAT-3’
*PPIA* Reverse: 5’-TCTTTCACTTTGCCAAACACC-3’

### Statistical analysis

Experiments were performed for an n ≥ 3 independent trials as indicated in figure legends. One-way ANOVAs were performed with a post-hoc Bonferroni correction for multiple comparisons. t-tests were performed where only one comparison was performed. A p-value <0.05 was accepted to be a statistically significant difference between groups. Data were analyzed using GraphPad Software Version 6.0. Data are expressed as means ± standard deviation (SD).

### Data deposition

All raw data used for figures and analysis is deposited in S1 File.

## Results

### HDM exposure, but not cigarette smoke, induces uric acid production *in vivo* and *in vitro*

**Fig 1:**
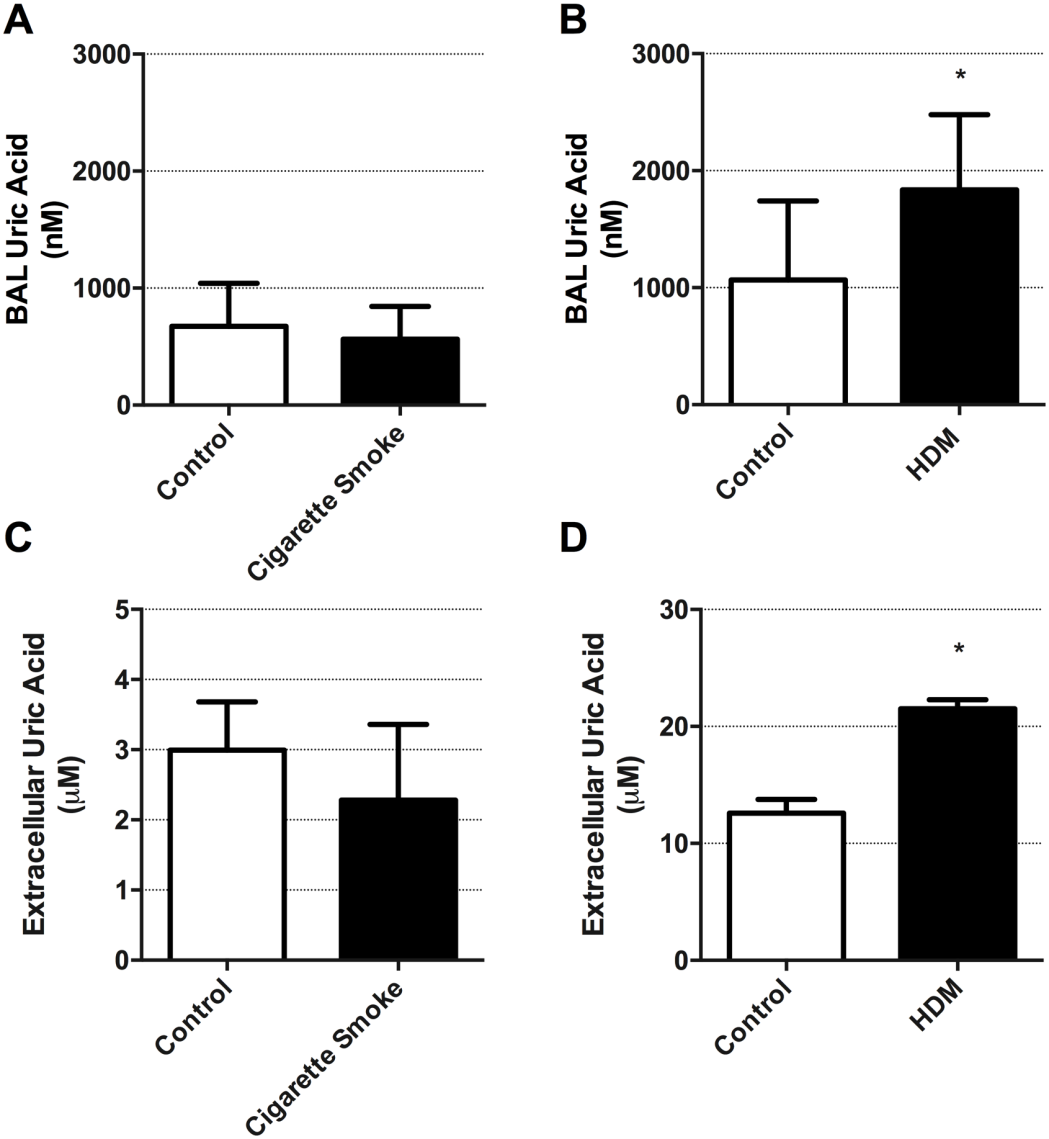
Exposure to HDM but not cigarette smoke induces uric acid production *in vivo* in mouse lungs and *in vitro* in human airway epithelial cells. (**A**) BAL analysis of uric acid levels in mice 24h following exposure to air or cigarette smoke (n = 10). (**B**) BAL analysis of uric acid levels in mice 24h following exposure to PBS or HDM (n = 10). (**C**) HBEC-6KT human airway epithelial cell culture supernatant analysis of uric acid levels 24h following exposure to control (media alone) or cigarette smoke (4% cigarette smoke extract conditioned media) (n = 6). (**D**) HBEC-6KT human airway epithelial cell culture supernatant analysis of uric acid levels 24h following exposure to control (PBS) or HDM (50 μg/ml) (n = 6). Data represent mean +/- SD. Significance is represented by * = p<0.05 relative to control.

We have previously demonstrated that uric acid levels are elevated in BAL from mouse models of allergic airways disease and may be important in asthma pathogenesis[26]. It remains unknown if elevations in lung uric acid levels are important in other chronic respiratory diseases including COPD. We therefore examined if cigarette smoke exposure induced uric acid elevations *in vivo* and *in vitro* and compared this to induction by HDM. Using an established *in vivo* mouse model of cigarette smoke exposure for 8 weeks, we demonstrate that cigarette smoke fails to alter BAL uric acid levels (Fig 1A). In contrast, HDM exposure for 2 weeks increased BAL uric acid levels (Fig 1B, p<0.05). We analyzed BAL cell counts to confirm our exposures were robust and induced inflammatory responses within the lung. Cigarette smoke exposure induced an increase in total cells (33.6*10^5 cigarette smoke vs 6.7*10^5 control air—p<0.05) that was primarily associated with an increase in neutrophil (11*10^5 cigarette smoke vs 0.8*10^5 control air—p<0.05) and macrophages (18*10^5 cigarette smoke vs 6.5 control air—p<0.05). HDM exposure induced an increase in total cells (129.2*10^5 HDM vs 16.5*10^5 diluent—p<0.05). All cell differentials examined were increased including neutrophils (6.0*10^5 HDM vs 1.4*10^5 diluent—p<0.05), lymphocytes (9.5*10^5 HDM vs 1.0*10^5 diluent—p<0.05), macrophages (53.0*10^5 HDM vs 14.6*10^5 diluent—p<0.05), and eosinophils (60.7*10^5 HDM vs 3.0*10^5 diluent—p<0.05).

We next interrogated *in vitro* model systems with human airway epithelial cells. Similar to *in vivo* experiments, cigarette smoke exposure conditioned media failed to induce uric acid release from HBEC-6KT human airway epithelial cells (Fig 1C). In contrast, HDM (50 μg/ml) exposure increased uric acid release from HBEC-6KT human airway epithelial cells (Fig 1D, p<0.05), as we have previously shown[26]. We observed no significant increases in XDH gene expression following cigarette smoke extract exposure or HDM exposure (data not shown).

### Airway epithelial cells from asthmatics, but not COPD subjects, have elevated basal uric acid production

**Fig 2:**
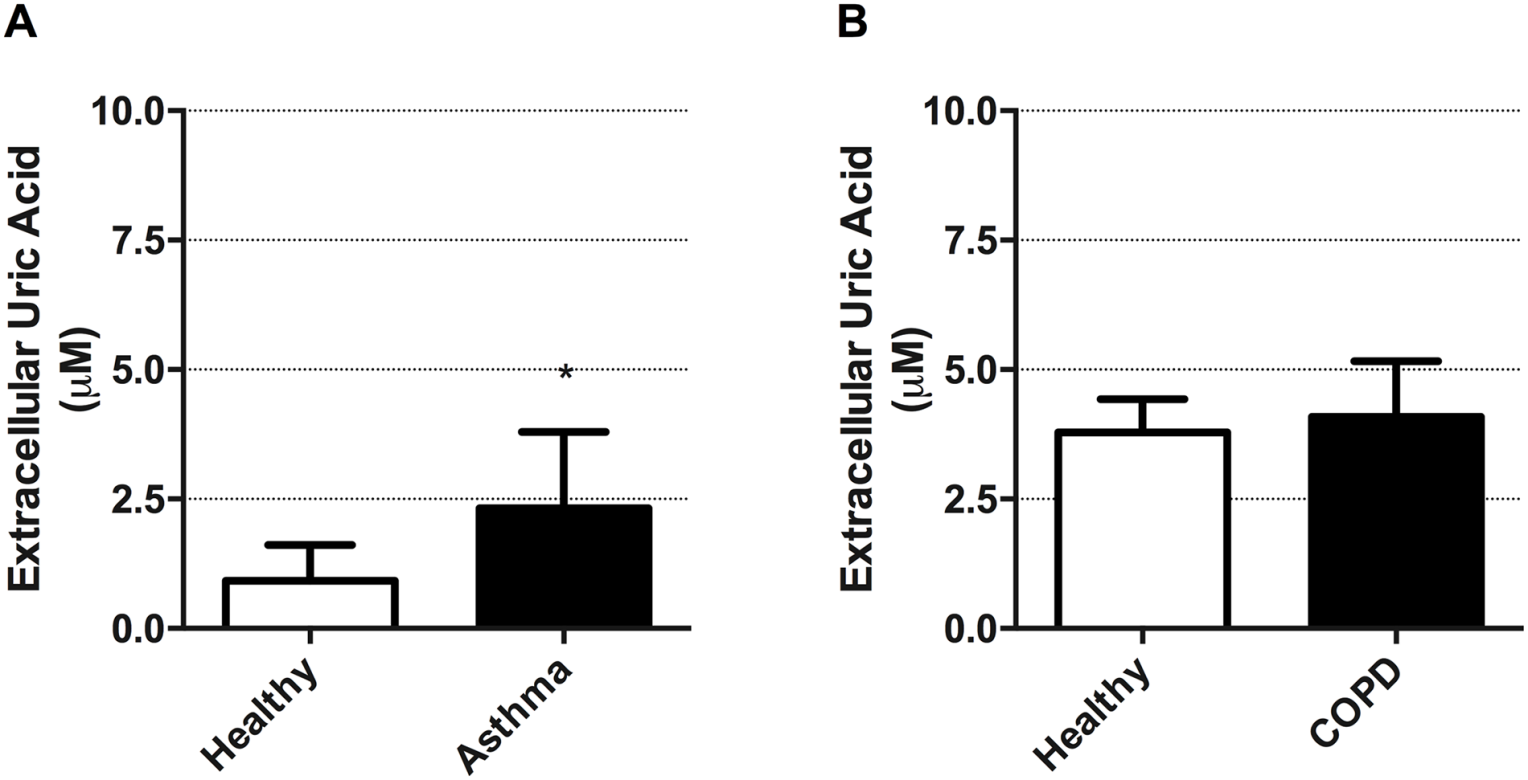
Primary airway epithelial cells from asthmatics, but not subjects with COPD, generate elevated extracellular uric acid levels. (**A**) Cell culture supernatant analysis of uric acid levels in un-stimulated primary human airway epithelial cells from healthy age-matched control subjects and asthmatics following 24h of culture. (**B**) Cell culture supernatant analysis of uric acid levels in un-stimulated primary human airway epithelial cells from healthy age-matched control subjects and those with COPD following 24h of culture. Data represent mean +/- SD, n = 5–8. Significance is represented by *p<0.05 relative to healthy controls.

We next explored if primary human airway epithelial cells from individuals with asthma and COPD differed in basal release of uric acid relative to age-matched healthy controls. Clinical subject characteristics are listed in Table 1. We demonstrate that airway epithelial cells from asthmatics have intrinsically elevated levels of uric acid release relative to healthy controls (Fig 2A, p<0.05). In contrast, airway epithelial cells isolated from individuals with COPD did not demonstrate any intrinsic abnormalities in uric acid release relative to healthy controls (Fig 2B).

### Airway epithelial cell production and extracellular transport of uric acid is sensitive to allopurinol and MK-571 pharmacological interventions

**Fig 3:**
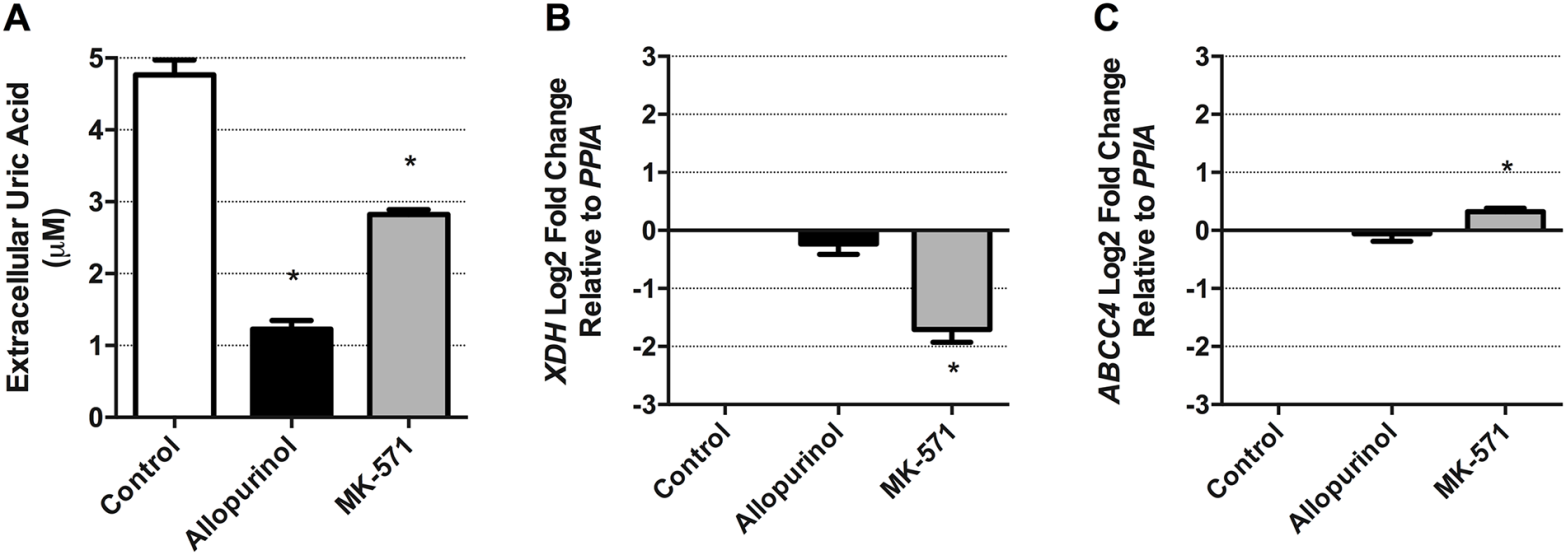
Extracellular uric acid levels from human airway epithelial cells can be attenuated with the XDH inhibitor, allopurinol, and the ABCC4 inhibitor, MK-571. **(A)** Allopurinol (500 μM) and MK-571 (20 μM) treatment for 24h attenuated un-stimulated production of uric acid from HBEC-6KT, a human airway epithelial cell line derived from a healthy individual. **(B)** *XDH* and **(C)** *ABCC4* gene expression in HBEC-6KT cells. Data represent mean +/- SD, n = 3. Significance is represented by * = p<0.05 relative to control.

XDH is the enzyme responsible for uric acid production [29, 30], while ABCC4 is capable of transporting uric acid to the extracellular space [26, 33, 67]. To confirm that uric acid production and extracellular transport are through conventional pathways we performed mechanistic experiments with allopurinol to inhibit XDH and MK-571 to inhibit ABCC4. Treatment of resting HBEC-6KT human airway epithelial cells with allopurinol (500 μM) or MK-571 (20 μM) for 24h attenuated extracellular levels of uric acid from cultured human airway epithelial cells (Fig 3, p<0.05). To determine if allopurinol and MK-571 have impacts at the transcriptional level on XDH and ABCC4, we determined gene expression in control unstimulated cells. Allopurinol had no impact on *XDH* or *ABCC4* gene expression. In contrast, MK-571 attenuated *XDH* gene expression and augmented *ABCC4* gene expression (Fig 3B–3C).

### Extracellular uric acid levels in cultured human airway epithelial cells following chemical stimulation

**Fig 4:**
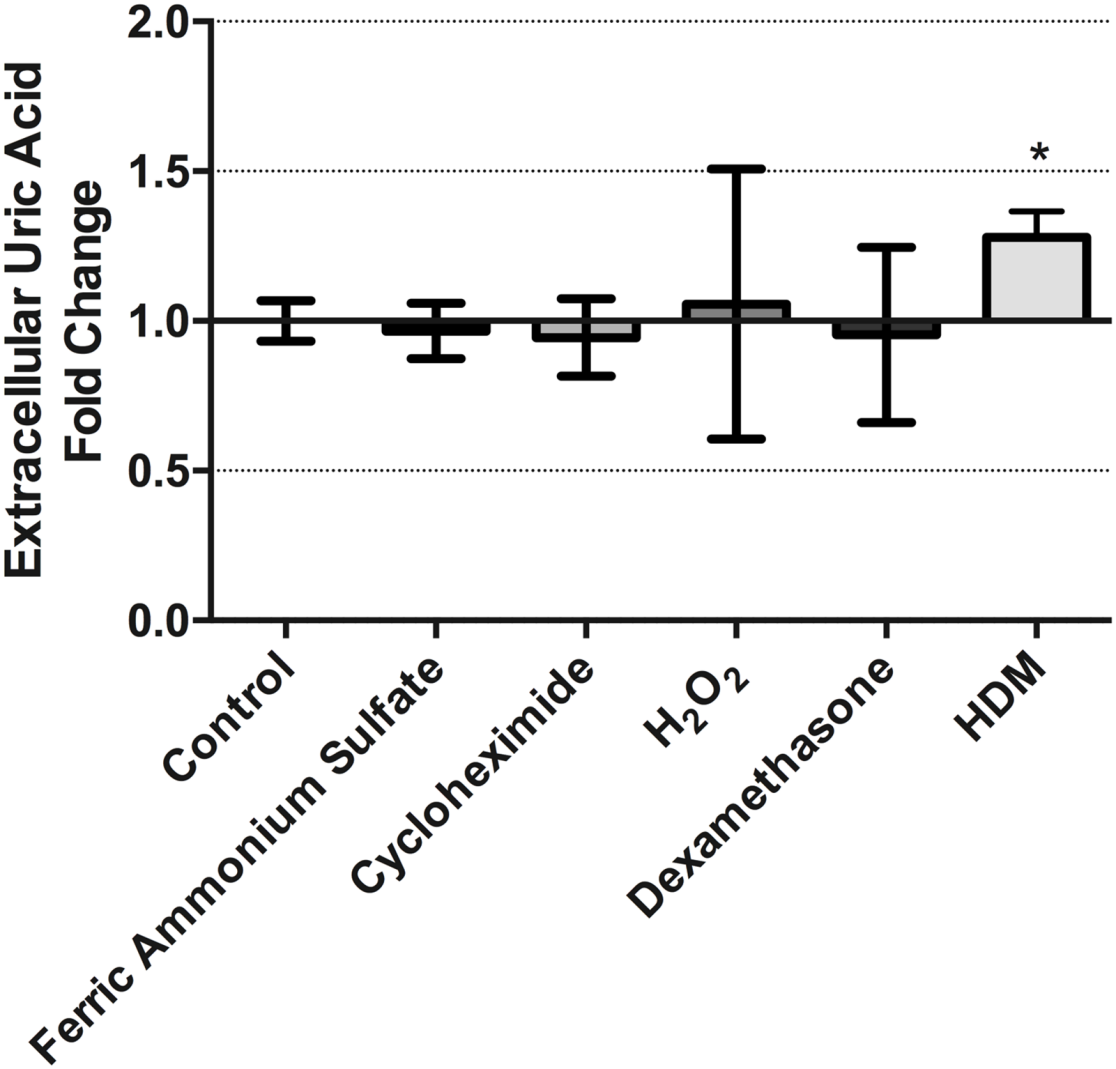
Extracellular uric acid levels in cultured human airway epithelial cells are not augmented by diverse chemical exposures. Treatment of HBEC-6KT human airway epithelial cells with ferric ammonium sulfate (50 μM), cycloheximide (0.01 μg/ml), H_2_O_2_ (100 μM), and dexamethasone (10 μg/ml) for 24h did not induce elevations in extracellular uric acid levels in contrast to the positive control of HDM (50 μg/ml). Data represent mean +/- SD, n = 3. Significance is represented by * = p<0.05 relative to control.

Diverse chemical compounds have been used to induce XDH activity in a variety of cell types[44–48, 68]. We therefore explored whether known chemical inducers of XDH activity were associated with increased extracellular levels of uric acid in cultures of HBEC-6KT human airway epithelial cells. Treatment of HBEC-6KT cells with ferric ammonium sulfate (50μM), cycloheximide (0.01 μg/ml), H_2_O_2_ (100 μM), and dexamethasone (10 μg/ml) for 24h all failed to increase elevations in extracellular uric acid levels in contrast to the positive control of HDM (50 μg/ml) (Fig 4).

### The inflammatory mediators, TNF-α and IFN-γ, induce uric acid production and *XDH* gene expression in human airway epithelial cells

**Fig 5:**
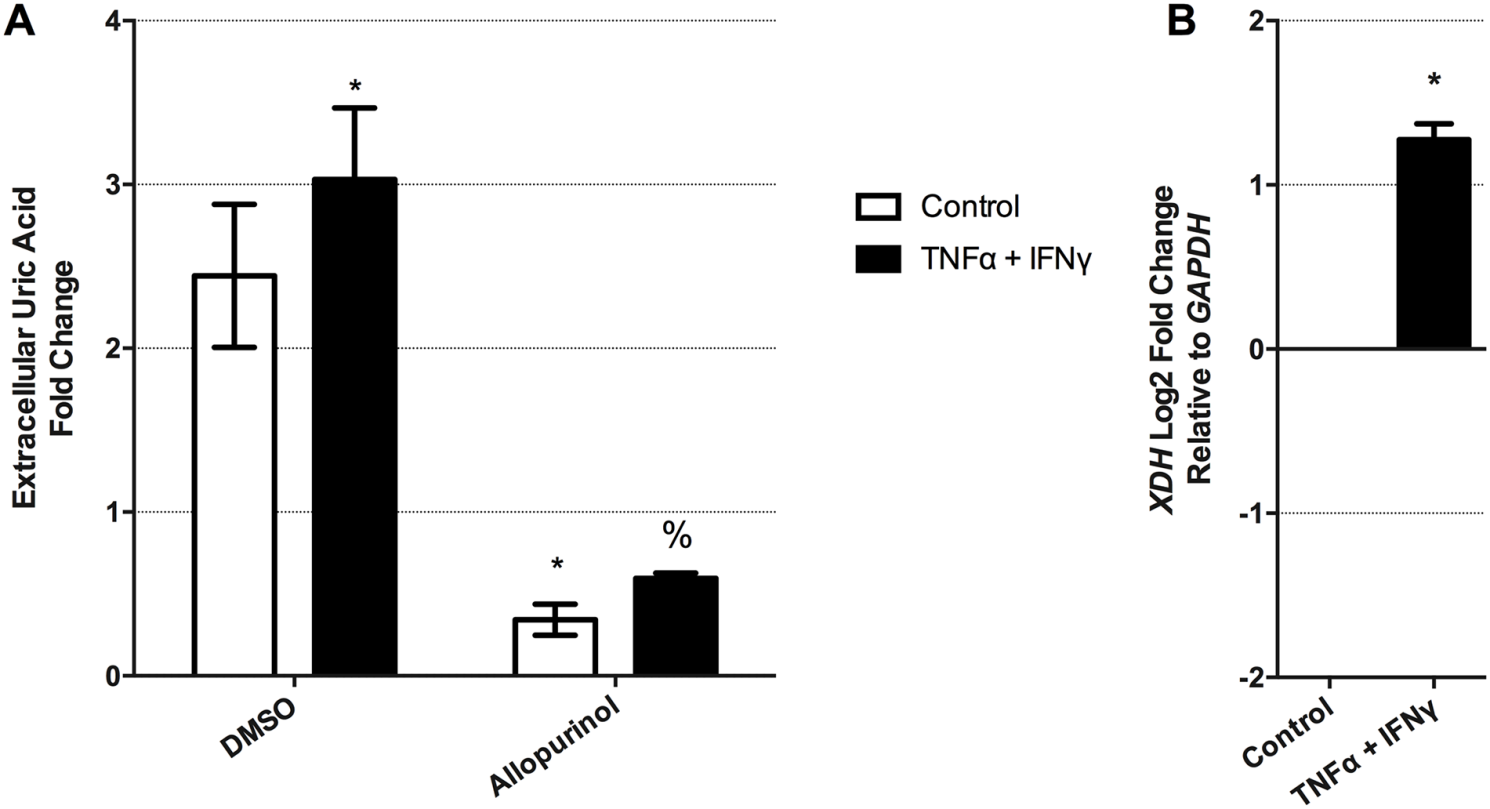
TNF-α and IFN-γ elevate extracellular uric acid levels and XDH gene expression in human airway epithelial cells. **(A)** HBEC-6KT human airway epithelial cells were treated with TNF-α (20ng/ml) and IFN-γ (1000U/ml) for 24h followed by analysis of cell culture supernatant for uric acid levels in the presence or absence of allopurinol (500 μM) **(B)** *XDH* gene expression following TNF-α (20ng/ml) and IFN-γ (1000U/ml) treatment for 24h. Data represent mean +/- SD, n = 3. Significance is represented by * = p<0.05 relative to DMSO/control, % = p<0.05 relative to DMSO/ TNF-α+IFN-γ

Elevations in uric acid and subsequent formation of crystals are danger signals during early inflammatory events[35, 69, 70]. However, it is unknown if regulation of uric acid production occurs during early inflammatory events. To investigate this, we examined whether pro-inflammatory conditions could stimulate uric acid production in human airway epithelial cells, using TNF-α (20ng/ml) and IFN-γ (1000U/ml) as positive control stimulus based on previous reports[46]. The combination of TNF-α (20ng/ml) and IFN-γ (1000U/ml), induced an increase in the production of uric acid from HBEC-6KT human airway epithelial cells, and this could be attenuated by the inhibition of XDH with allopurinol (500 μM) (Fig 5A, p<0.05). Associated with the increase in uric acid production, TNF-α and IFN-γ also induced an increase in *XDH* gene expression (Fig 5B, p<0.05). Administration of TNF-α or IFN-γ alone failed to induce any changes in extracellular uric acid levels or *XDH* gene expression (data not shown). IL-6 administration had no impact on extracellular uric acid levels or XDH gene expression (S1 Fig).

### EGF supplementation in cell culture media does not induce XDH gene expression or elevations in extracellular uric acid production

*In vitro* experiments with human airway epithelial cells commonly use cell culture media containing EGF at varying concentrations. EGF can upregulate *XDH* gene expression in L2 rat lung epithelial cells and may be a confounding variable in our *in vitro* experimental designs[71]. We therefore determined if a 24h incubation with a three-log10 dose range of EGF could influence extracellular uric acid levels and *XDH* gene expression in HBEC-6KT human airway epithelial cells and compared this to exposure to the standard 0.6ng/ml concentration we use throughout our experiments. We failed to observe increases in uric acid production or increase in *XDH* gene expression with EGF at concentrations ranging from 0.1ng/ml to 10ng/ml (S2 Fig).

## Discussion

The presence of uric acid in lungs may provide an antioxidant capable of protecting from inhaled environmental insults while simultaneously functioning as an alarm for the immune system. We set out to explore how airway epithelial cells regulate extracellular uric acid production. We demonstrate that exposure to HDM, but not cigarette smoke, stimulates uric acid production *in vivo* and *in vitro*. We next show that airway epithelial cells from asthma, but not COPD patients, produce elevated levels of extracellular uric acid in culture under basal conditions. We then show that extracellular uric acid levels from airway epithelial cells are produced by XDH and transported by ABCC4. Lastly, we demonstrate that the pro-inflammatory cytokine combination of TNF-α and IFN-γ elevate extracellular uric acid levels and *XDH* gene expression in human airway epithelial cells. Collectively, our results suggest that active production of uric acid from human airway epithelial cells may be intrinsically altered in asthma and may be further induced by pro-inflammatory cytokines. Precisely how elevated airway uric acid levels contribute to prevention or development of chronic respiratory diseases requires further study.

Exposure to allergens and particulate matter are important risk factors for the development and exacerbation of many chronic respiratory diseases. Airway epithelial cells respond to allergens and particulate matter by secreting a variety of immune mediators[1–5]. Human exposure models with allergens and particulate matter induce uric acid production in asthmatics and healthy controls, respectively[28, 43], that may be produced in part from airway epithelial cells[26]. Cigarette smoke is another clinically important risk factor for chronic respiratory disease. To examine whether cigarette smoke exposure induced uric acid similar to allergen and particulate matter exposure, we performed *in vivo* and *in vitro* cigarette smoke exposure experiments. We used an established *in vivo* model of chronic cigarette smoke exposure (5 days per week for 8 weeks) that represents exposure in a pack-a-day human smoker[57]. This model displays the hallmark disease features of neutrophilia, emphysema-like alveolar enlargement and changes in lung mechanics [53]. However, we failed to detect elevations in airway uric acid levels. We explored alternative durations of smoking ranging from 1wk to 12wks and also failed to see elevations in uric acid at any other timepoint (data not shown). To directly interrogate whether cigarette smoke exposed airway epithelial cells produced uric acid, we performed *in vitro* experiments with cigarette smoke extract conditioned media[49, 50]. Consistent with our *in vivo* experiments, *in vitro* exposure to cigarette smoke did not elevate extracellular uric acid levels. The data suggest that airway epithelial cell production of uric acid in response to environmental exposures is not a generalized phenomenon[26] and does not occur with cigarette smoke.

Intrinsic abnormalities in airway epithelial cells are observed in chronic respiratory diseases including asthma, COPD, and CF [8–12, 15–17]. It therefore remained possible that intrinsic abnormalities that impact uric acid production (i.e. XDH expression and activity) and extracellular transport (i.e. ABCC4 expression and activity) could be present in airway epithelial cells from specific disease phenotypes. To explore this possibility we collected primary human airway epithelial cells from asthmatics and subjects with COPD and compared each of these cohorts to appropriately age-matched healthy controls. We demonstrate intrinsic elevations in extracellular uric acid from airway epithelial cells from asthmatics, but not from those with COPD. A limitation of this observational study is that we were unable to perform mechanistic experiments to explore if the intrinsic abnormalities in cells from asthmatics were dependent on the function of XDH or ABCC4. To address this possibility, we performed an intervention with the XDH inhibitor, allopurinol, and the ABCC4 inhibitor, MK-571 in a human airway epithelial cell line from a healthy individual (HBEC-6KT), demonstrating that these two pathways are important for regulating extracellular levels of uric acid. Future studies using different patient populations (e.g. well-controlled vs un-controlled severe asthmatics[72]) are required to confirm our findings of intrinsic abnormalities in uric acid production from airway epithelial cells isolated from asthmatics. The use of pharmacological inhibitors would determine the contributions of XDH and ABCC4 activity to this observation in asthmatics.

XDH and ABCC4 have historically been pharmacologically targeted at the protein level with allopurinol and MK-571, respectively. To determine if allopurinol and MK-571 have impacts at the transcriptional level on XDH and ABCC4, we determined gene expression in control unstimulated cells. Our data demonstrates that allopurinol does not regulate XDH or ABCC4 gene expression, supporting the proposed mechanism of action of allopurinol for direct inhibition of XDH protein function. In contrast, MK-571 had a surprising impact on down-regulation of XDH gene expression and up-regulation of ABCC4 gene expression. These results suggest that the mechanism by which MK-571 inhibits uric acid transport to the extracellular space is likely to be more complex than originally anticipated[26] and involve both protein inhibition and alterations of genes in this pathway at the transcriptional level. For these reasons, caution should be used when interpreting the functional outcomes of MK-571 application as an inhibitor of ABCC4-mediated uric acid transport in human airway epithelial cells.

XDH expression and activity can be induced by diverse chemical stimuli including ferric ammonium sulfate, cycloheximide, hydrogen peroxide, and dexamethasone[44–48, 68], although these chemicals have not been assessed for the responses they induce in human airway epithelial cells. Following a 24h incubation period, we did not observe any increase in extracellular uric acid from human airway epithelial cells under any experimental condition, despite observing an increase with our positive control of HDM. We observed no changes in cell morphology that would indicate the occurance of cell death (data not shown) for any of the experimental conditions. A limitation of our study is the examination of extracellular uric acid levels as a surrogate for intracellular changes in XDH expression and activity. We decided to quantify extracellular uric acid since this is consistent with related studies examining this compartment in human, mouse, and *in vitro* models [25, 26, 28, 41–43] that have explored the antioxidant and DAMP activities of uric acid. It remains possible that increased *XDH* gene and protein expression occurs in response to each chemical stimuli assessed, with intracellular uric acid concentrations being elevated without extracellular release.

Previous reports have demonstrated that the pro-inflammatory cytokines TNF-α and IFN-γ were able to induce XDH activity in a Madin-Darby bovine kidney tubular epithelial cells following 24h incubation[46]. We explored whether this mechanism was conserved in human airway epithelial cells and showed that exposure to TNF-α and IFN-γ did increase extracellular uric acid levels and *XDH* gene expression. Consistent with our experiments in un-stimulated human airway epithelial cells, our data again implicated XDH and ABCC4 in this biological process. The implications of pro-inflammatory cytokine induction of uric acid production by airway epithelial cells may be both beneficial and harmful, due to the antioxidant and DAMP nature of this molecule. In the context of heightened inflammation and recruitment of inflammatory cells, oxidative stress may be elevated and the presence of uric acid could scavenge free radicals[36–38]. Alternatively, the elevations in uric acid may lead to further recruitment of inflammatory cells and activation of adaptive immune responses[25, 34, 35]. We suggest that future studies aimed at reconciling the multiple biological activities of uric acid during inflammatory events will need to consider the kinetics of uric acid production, extracellular transport, and concentrations in microenvironments, similar to studies exploring cyclic AMP compartmentalization and regulation[73]. Furthermore, interrogating how other cytokine combinations that are frequently associated with the asthma phenotype (e.g. IL-13, IL-4, TSLP) may reveal novel mechanisms for regulating XDH expression and uric acid production.

In closing, using *in vivo*, *in vitro*, and clinical samples, we explored the overarching hypothesis that uric acid production by airway epithelial cells can be induced by stimuli associated with chronic respiratory diseases. We demonstrate that exposure to the allergen, HDM, induces uric acid production from human airway epithelial cells and that asthmatics have an elevated intrinsic production of uric acid. These findings were not observed in airway epithelial cells collected from individuals with a history of cigarette smoking or with *in vitro* or *in vivo* cigarette smoke exposure models. An elevation in uric acid production induced by TNF-α and IFN-γ is associated with an increase in *XDH* gene expression and is sensitive to the XDH inhibitor, allopurinol, and the ABCC4 inhibitor, MK-571. Collectively, our results suggest that active production of uric acid from human airway epithelial cells may be intrinsically altered in asthma and may be further induced by pro-inflammatory cytokines. In the future, a deeper exploration into production kinetics, extracellular transport, and microenvironments will help uncover the diverse biological roles of uric acid in lung health and disease.

## Supporting information

S1 Fig IL-6 does not elevate extracellular uric acid levels or *XDH* gene expression in HBEC-6KT human airway epithelial cells. Human airway epithelial cells were exposed to recombinant human IL-6 (100ng/ml) for 24h followed by analysis of **(A)** cell culture supernatant uric acid levels and **(B)** *XDH* gene expression. Data represent mean +/- SD, n = 3. Significance is represented by * = p <0.05 relative to controls.
(TIFF)

S2 Fig EGF does not elevate extracellular uric acid levels or *XDH* gene expression in HBEC-6KT human airway epithelial cells. Human airway epithelial cells were exposed to increasing concentrations of EGF or HDM in culture for 24h followed by analysis of **(A)** cell culture supernatant uric acid levels and **(B)** *XDH* gene expression. Data represent mean +/- SD, n = 3. Significance is represented by * = p <0.05 relative to controls.
(TIFF)

S1 File Excel file of all raw data used for figures and analysis. (XLSX)
