# Supplementary figures and images for: Regulation of xanthine dehydrogensase gene expression and uric acid production in human airway epithelial cells
Source: PLoS One. 2017 Sep 1;12(9):e0184260. doi: 10.1371/journal.pone.0184260 (PMC5580912; doi:10.1371/journal.pone.0184260)

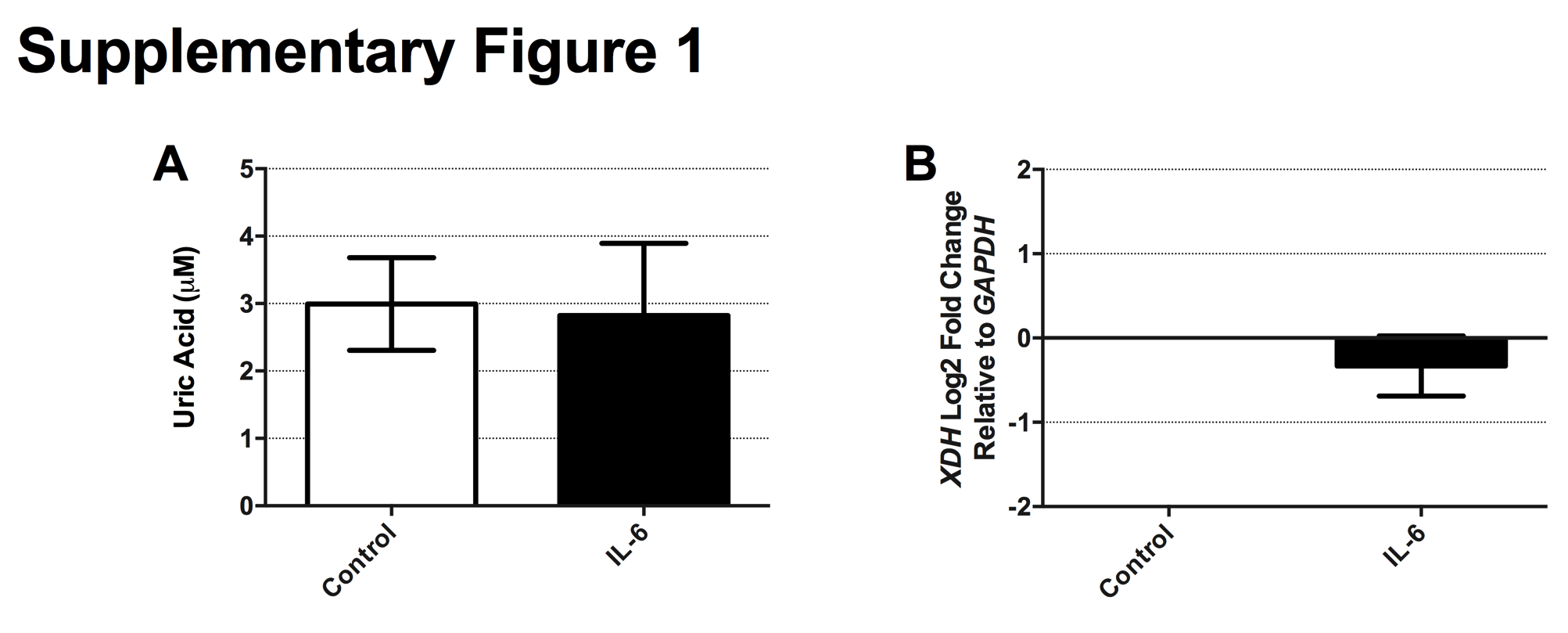

Supplement: S1 Fig — Human airway epithelial cells were exposed to recombinant human IL-6 (100ng/ml) for 24h followed by analysis of (A) cell culture supernatant uric acid levels and (B) XDH gene expression. Data represent mean +/- SD, n = 3. Significance is represented by * = p <0.05 relative to controls. (TIFF) [file pone.0184260.s001.tiff]

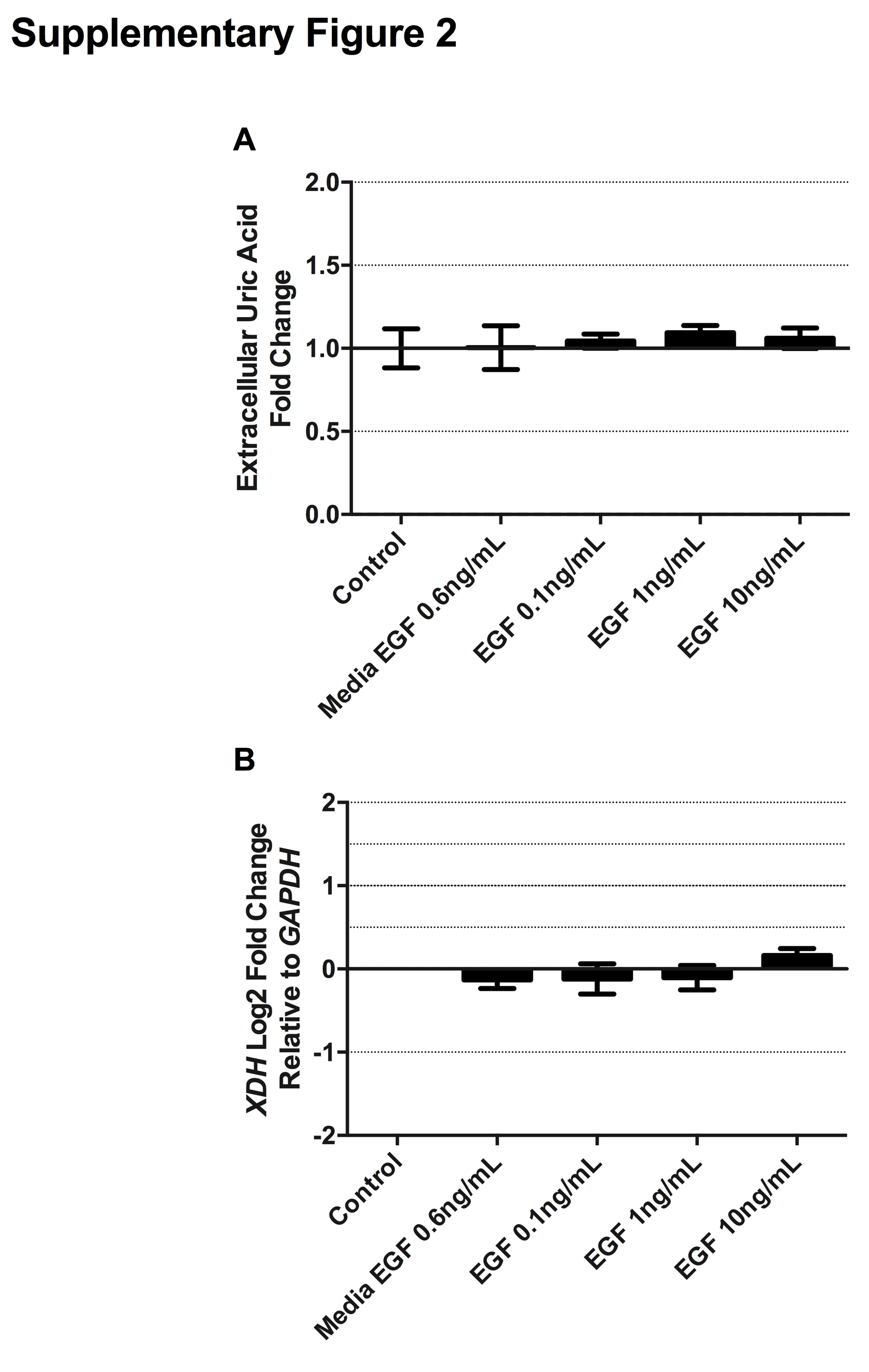

Supplement: S2 Fig — Human airway epithelial cells were exposed to increasing concentrations of EGF or HDM in culture for 24h followed by analysis of (A) cell culture supernatant uric acid levels and (B) XDH gene expression. Data represent mean +/- SD, n = 3. Significance is represented by * = p <0.05 relative to controls. (TIFF) [file pone.0184260.s002.tiff]
